# Personalized Management in Low-Risk Prostate Cancer: The Role of Biomarkers

**DOI:** 10.1155/2012/327104

**Published:** 2012-12-13

**Authors:** Siebren Dijkstra, Agus Rizal A. H. Hamid, Gisèle H. J. M. Leyten, Jack A. Schalken

**Affiliations:** ^1^Department of Urology, Radboud University Nijmegen Medical Centre, Geert-Grooteplein Zuid 10, P.O. Box 9101, 6500 HB Nijmegen, The Netherlands; ^2^Department of Urology, Dr. Cipto Mangunkusumo Hospital, Faculty of Medicine, University of Indonesia, Jl. Diponegoro no. 71, Jakarta 10430, Indonesia

## Abstract

Current criteria to predict low-risk prostate cancer (PCa) are still subject to discussion as a substantial number of PCa patients who progress to a more aggressive disease seem to be missed, using these criteria. The main challenge in PCa diagnosis, therefore, is to distinguish patients with low-risk PCa who will show slow progression of disease from patients at risk for progression to a more aggressive cancer. The current discovered biomarkers could potentially guide in this management and improve detection, staging, and prognosis. This paper provides an overview of the current available serum-, urine-, and tissue-based biomarkers in PCa and evaluates the clinical usefulness of these biomarkers in the detection and management of low-risk PCa.

## 1. Introduction

Prostate cancer (PCa) is the most frequently diagnosed malignancy among men in the Western society. The lifetime probability of developing PCa is 16.5% and the risk of death due to PCa is 1 in 30 [1]. The introduction and widespread use of serum prostate-specific antigen (PSA) in the late 1980s led to a considerable increase in PCa incidence, and currently this is still the most common screening method for PCa. However, low specificity of PSA screening for PCa detection leads to an increase in diagnostic prostate biopsies, which in turn results in diagnosis of many tumors which would not have become clinically significant during lifetime [2]. Therefore, PCa screening based on serum PSA levels remains a controversy, as these insignificant tumors are a psychological burden to patients and provide an increase in healthcare costs for the community [2–4].

The fraction of screen-detected cancers that would not have been diagnosed in the absence of screening is defined as overdiagnosis and might lead to unnecessary treatment [5]. Rates of overdiagnosis estimates, using PSA screening, range between 42% and 66% [6]. Therefore, the main challenge in PCa diagnosis is to distinguish patients with low-risk PCa who will show slow progression of disease (potential candidates for active surveillance) from patients at risk for progression to a more aggressive cancer (candidates for additional treatment). Current definitions for low-risk PCa show a substantial risk of cancer misclassification, and therefore, better prediction tools are urgently needed. In this paper, we discuss the definition of low-risk PCa and the role of biomarkers in the diagnosis of PCa and management of active surveillance. 

## 2. Definition of Insignificant PCa

The ideal, noninvasive management for insignificant PCa patients is active surveillance. However, active surveillance can only be applied to patients with a minimum risk of disease progression. With the current criteria, there still is a substantial risk of misclassification, and therefore, the definition of insignificant PCa is subject to discussion [7]. Criteria to predict insignificant PCa based on prostate biopsy outcome were first described by Epstein et al. based on a series of 157 consecutive radical prostatectomies for cancers detected by a five-core biopsy procedure [8]. These criteria include clinical stage T1c, PSA density <0.15 ng/mL, no Gleason pattern 4 or 5, <3 positive cores, and <50% cancer per core [8]. According to these criteria, pathologically confirmed insignificant PCa may be expected to be correctly predicted in 73% of patients, meaning 27% of patients is underdiagnosed [7]. Since the establishment of these criteria, numerous studies reported additional selection criteria for predictive models with an accuracy ranging from 73% to 79%, similar results compared to the Epstein criteria [7, 9–11]. However, Chun et al. developed a nomogram based on a cohort of 1132 men using PSA level, clinical stage, biopsy Gleason sum, core cancer length, and percentage of positive cores, finding a predictive accuracy of 90% [12]. The diversity in definitions and outcome in studies on the criteria for insignificant PCa reflects the difficulty to correctly predict insignificant PCa, and with the current available models, still 10% to 20% of predictions are incorrect [12]. Decreasing this number by identification of these patients could be realized by stricter follow-up programs and undergoing more regular or high-density biopsy sessions. This would, however, lead to an expansion in healthcare costs and a burden to the patient. A more patient-friendly, less invasive, and potentially better way to identify these patients might be found in novel PCa-specific biomarkers.

The last decade tremendous progress has been made in the field of molecular profiling, resulting in new breakthroughs. This led to the discovery of novel biomarkers which could potentially aid in personalized management by accurately predicting the biological behavior.

## 3. Biomarker Substrates

A biomarker can be defined as a characteristic that is objectively measured and evaluated as an indicator of normal biological processes, pathogenic processes, or pharmacologic responses to a therapeutic intervention [13]. Ideally, a new PCa biomarker tool should meet the following criteria: it should be a noninvasive test, produced by tumor tissue only and have the ability to detect PCa in an early stage. Thereby, it should be able to differentiate aggressive tumors from insignificant tumors with a high specificity and sensitivity and be as inexpensive as possible to encourage widespread use. Three distinctive groups can be categorized as substrates for PCa biomarker analysis: blood, urine, and tissue.

## 4. Blood Markers

### 4.1. Prostate-Specific Antigen

PSA was approved by the US Food and Drug Administration (FDA) in 1994 as a diagnostic marker and is currently the most widely used, blood-derived prostate-specific biomarker. Although PSA is used as a screening tool to detect PCa, it is not a cancer-specific marker. PSA, also known as kallikrein 3, is expressed by both normal and neoplastic prostate epithelial tissue [14]. Generally, under normal conditions, PSA blood levels are low. However, certain circumstances can cause a rise in PSA blood levels, for example, benign prostatic hypertrophy, prostatitis, and PCa. Due to its low specificity for PCa, prostate biopsies based on PSA values >4.0 ng/mL lead to negative biopsies in approximately 70% of the patients [15, 16]. For PSA levels between 4.0 and 10.0 ng/mL, the positive predictive value is only 25% [15]. In the majority of evaluated patients, no cancer will be detected. These people, however, are exposed to potential biopsy-related events such as bleeding, urinary obstruction, and infections. Most of these men even remain suspicious and will undergo further prostatic evaluations. Furthermore, from the patients that are diagnosed with PCa, a substantial amount of the cancers are considered to be insignificant and are thus overdiagnosed [6]. An ERSPC study demonstrated that one would need 1055 men to screen and 37 cases to treat to prevent one PCa death over 11 years [17]. In order to attempt to overcome these PSA limitations, numerous PSA-related strategies have been evaluated.

### 4.2. Prostate-Specific Antigen Kinetics

These PSA derivatives have been evaluated in the attempt to improve the diagnostic accuracy of total PSA and the additional prognostic value for patients in active surveillance. Although PSA kinetics are easy to apply, several studies that relate PSA velocity (absolute increase or decrease of PSA in a certain time period) and PSA doubling time (the time interval for PSA to double in value) with outcome during active surveillance are based on small numbers, have limited follow-up time, and show contradictory results [18]. Although PSA kinetics can be critical for understanding prognosis in advanced or relapsed PCa, there is no justification for the use of PSA kinetics in clinical decision making in early-stage PCa [19, 20].

### 4.3. Molecular Prostate-Specific Antigen Forms

PSA circulates in the blood in two general forms: a complexed form (attached to proteins) and an unbound form. It has been demonstrated that a lower value of the “free PSA/total PSA” ratio is correlated with a higher probability of finding PCa on biopsy; however, as with PSA, there is no cutoff that completely discriminates PCa from normal tissue [21, 22]. The prognostic value of the free/total PSA ratio in active surveillance protocol is still unknown and remains to be unraveled. 

[-2]ProPSA, a specific PSA isoform, has been demonstrated to significantly outperform the use of total PSA and percent of free PSA alone [23–25]. Using [-2]ProPSA in combination with PSA and free PSA, a mathematical formula can be generated (prostate health index [*phi*]; [-2]ProPSA/free PSA × PSA^1/2^) [24]. In their study, Catalona et al. described that the relative risk of Gleason score ≥7 was increased at higher phi scores [24]. In a study by Guazzoni et al., they found phi to be a strong predictor of PCa at initial extended biopsies; however, it did not improve the prediction of Gleason score ≥7 [25]. Therefore, the clinical applicability of this recent FDA-approved diagnostic test in low-risk PCa needs further research.

### 4.4. Circulating Tumor Cells (CTCs)

CTCs have been described for the first time in 1869 in a man with metastatic disease. It is because of recent advances in technology that a reliable method to isolate and enumerate CTCs from the blood has been developed. The presence of CTCs and the number of CTCs in the blood are shown to be associated with overall survival in castration-resistant PCa patients [26–28]. Although CTCs can also be detected in some patients at the time of PCa diagnosis, the prognostic significance in those patients remains unclear. Therefore, there seems to be no role for CTCs in PCa diagnosis or management of insignificant PCa.

## 5. Urine Markers

Because of the anatomical location of the prostate and the direct connection to the urethra, urine can serve as an easy-to-obtain substrate to measure biochemical processes within the prostate. Since the expression of biomarkers in first-catch urinary samples is demonstrated to be higher after performing a digital rectal examination (DRE), this is recommended for every PCa biomarker-related urinary test [29–32].

### 5.1. Prostate Cancer Antigen 3 (PCA3)

In 1999 the DD3 gene was identified as a prostate-specific noncoding RNA found to be highly overexpressed in PCa tissue compared with normal or benign hyperplastic prostate tissue and currently better known as PCA3 [33]. After several clinical studies confirmed this finding, a commercial PCA3 urinary test became available (PROGENSA). To date PCA3 has been extensively studied for guiding biopsy decisions in men with PSA levels in the “gray area” (2.5 to 10.0 ng/mL) and for patients with previous negative biopsies and persistent elevated PSA levels. The test received the Conformité Européenne (EC) approval to assist in decision making for initial and repeat biopsy indications in 2006 and FDA approval for decision making in repeat biopsy indications in 2012. The PCA3 score is calculated as PCA3 mRNA/PSA mRNA × 1000. The FDA determined a cut-off value of 25 to be the most accurate. However, several studies show highest diagnostic performance rates with a cut-off value of 35 [34, 35]. Due to different results in various studies, this cut-off value is still subject to discussion.

A recent European randomized study of screening for prostate cancer (ERSPC) studied PCA3 as an initial diagnostic test. This study showed that PCA3 with a cut-off value of 35 had a sensitivity of 68.0% and specificity of 55.7% for the detection of PCa, compared to 57.4% and 53.8% for PSA (≥2 ng/mL) [36]. 

In active surveillance, PCA3 score was prospectively studied by Ploussard et al. in 106 low-risk PCa patients who underwent radical prostatectomy. They described a significant linear correlation between PCA3 and tumor volume for which it may be a useful marker to improve the selection for active surveillance [37]. However, PCA3 seems not to be correlated with extracapsular extension (ECE) and seminal vesicle invasion. Although higher PCA3 scores were associated with aggressive disease, the addition of PCA3 to aggressive PCa models did not improve prediction rates [38]. According to these studies, there seems to be no evidence for the usefulness of PCA3 in active surveillance protocols so far, as the test is not able to predict PCa aggressiveness.

### 5.2. Gene Fusions: TMPRSS2-ERG

The discovery of the *ETS *family transcription factor in 2005 using the cancer outlier profile analysis (COPA) led to a bioinformatic algorithm change in the field of PCa biology and biomarkers. The fusion between the ETS-related gene ERG and the transmembrane protease, serine 2 (TMPRSS2), has been found in approximately 50% of the PSA-screened prostate adenocarcinomas [39]. Nowadays, using gene fusions as a diagnostic PCa biomarker in urine is close to a clinical setting with a sensitivity of 30–50% and a specificity >90% in PSA-screened cohorts [40]. Based on the urine PCA3 score, the novel gene fusion expression score in urine, using transcription-mediated amplification (TMA) assay has been developed. So far, only one study used this calculation as a PCa diagnostic and prognostic marker. The study showed that higher gene fusion scores were associated with indicators of clinically significant cancer at biopsy outcome and prostatectomy [41]. More extensive research needs to be done on this marker assay to implement its role in diagnosis and prognosis of PCa.

### 5.3. Prostate Cancer Antigen 3 and Gene Fusion Panel

As most of the prostate cancers are multifocal, with each tumor presenting its own characteristics, it could be plausible to assume that a panel of cancer-related biomarkers is needed for more accurate diagnosis and prognosis.

The combination of PCA3 and gene fusion in urine, using the RUO technology, improves the prediction of PCa presence upon biopsy [42]. A recent study using the novel TMA-based urine assay showed that combining PCA3 and gene fusion results has markedly different risks of cancer, high-grade cancer, and clinically significant cancer upon biopsy. The combination scores may have additional utility to stratify a patient in common scenarios encountered in the early diagnosis of PCa. For example, men in the highest TMPRSS2-ERG + PCA3 score group enrolling in active surveillance have to be considered to get more extensive biopsies, because their risk of having significant disease that was undersampled on initial biopsy is high. The cut-off value in this study was based on quartiles of each score to stratify the patients [41].

### 5.4. microRNA (miRNA)

miRNAs are small non-protein-coding RNAs with a size of about 22 nucleotides, which are located within introns of coding or noncoding genes or within intergenic regions [43, 44]. MiRNAs might be potential markers for low-risk PCa detection, because they usually have a high stability in tissue (fixed) and body fluids and its expression changes according to the phases of prostate carcinogenesis (e.g., initiation versus progression versus metastasis) [45, 46]. Upregulation or downregulation of miRNAs has been reported to be related to the Gleason score, tumor stage, perineural invasion status, and biochemical progression of disease [43, 47]. However, the detection of these miRNAs mostly originated from tissue samples. The detection of miRNAs in blood and urine related to low-risk PCa has been explored. It has been shown that an upregulation of miR-141 and miR-375 expression in the blood has a correlation to PCa prognosis [48–50]. So far, only one study was able to detect miRNAs in urine (e.g., miR-107, miR-574-3p) and to potentially use them as a diagnostic marker [48].

### 5.5. Other Genes

Many other genes have been suggested to be useful as a potential marker for stratifying low-risk PCa patients, like engrailed-2 (EN2) [51], caveolin-1 (CAV-1) [52], secreted protein acidic and rich in cysteine-like 1 (SPARCL1) [53], and breast cancer anti-estrogen resistance protein 1 (BCAR1) [54].

## 6. Tissue Markers

Tissue markers can be a valuable resource once decisions have been made to either take a prostate biopsy or to surgically remove the prostatic gland. In active surveillance, this means information has been obtained from prostate biopsy sessions. A major drawback, however, is the reliability of the biopsies taken, as it is a random sample of the prostate and tumors might be missed.

The following tissue biomarkers have been repeatedly shown to be of prognostic value and are, therefore, assumed to be valuable prognostic markers. However, as most were retrospective studies, they are still research-use-only assays, and not (yet) used in daily practice.

### 6.1. Gene Fusions: TMPRSS2-ERG

Over 25 types of gene fusions in PCa tissue have been described so far [55]. Fusions between TMPRSS2 and ERG represent about 90% of all *ETS* gene fusions and are found to be highly specific for PCa [41, 56]. The difference of chromosomal aberrations, expression signatures, morphological features, and clinical outcomes between *ETS* gene fusion-positive and *ETS* gene fusion-negative PCa suggests that they are fundamentally different PCa classes [57]. The relation between PCa aggressiveness and the presence of gene fusion has been studied in various studies. Although two large observational studies described that the proportion of gene fusion presence was correlated with higher Gleason score, stage, and the incidence of metastases and PCa-related death, other studies show contradictory results on gene fusions as a prognostic marker [55, 58, 59].

### 6.2. Mitotic Index (Ki-67)

Expression of Ki-67 is strictly associated with cell proliferation and has been used in PCa since 1995 [60]. Ki-67 index is related to the Gleason score and low-risk PCa criteria [61]. Furthermore, Ki-67 index in biopsy core samples can be an independent factor to predict disease recurrence, regardless of the Gleason score [62]. One cohort study in clinically low-stage, low-grade, screen-detected PCa showed that Ki-67 was a significant predictor of PSA relapse after radical prostatectomy [63]. Furthermore, another study concerning low-risk PCa patients showed that a high Ki-67 can identify patients with poor outcome [64].

### 6.3. PTEN

PTEN (phosphatase and tensin homolog on chromosome 10) is one of the most frequently aberrant tumor suppressor genes and related to poor prognosis in PCa. This tumor suppressor gene is located on the chromosome 10q23 and plays a crucial role in carcinogenesis by antagonizing the PI-3K/Akt pathway and thereby promoting cell growth, proliferation, survival, and migration [65]. Two cohort studies detected PTEN expression at the first-time biopsy material and clinically localized PCa after radical prostatectomy and showed that loss of PTEN expression has higher risk of metastasis and recurrence, independent of prognostic clinicopathological factors [65, 66]. It means that loss of PTEN expression in early diagnosis can exclude PCa patients from low-risk PCa. A recent study showed that PTEN loss in combination with TMPRSS2-ERG fusion expression is correlated with a higher risk of poor prognosis [67]. 

### 6.4. E-Cadherin

Cadherins are a family of calcium-dependent adhesive molecules of which E-cadherin is the most prominent member. Cadherins are crucial in preserving epithelial cell-to-cell integrity [68]. Therefore, they are assumed to be involved in cancer progression to an invasive state. In PCa, a decreased expression of E-cadherin has been shown to be associated with a loss of tumor differentiation and poor prognosis [69–72].

### 6.5. EZH2

Increased expression of the EZH2 (enhancer of zeste homolog 2) gene has been observed in various aggressive tumors, that is, cancer of the prostate, breast, and bladder. The EZH2 gene encodes a polycomb-group (PcG) protein and is involved in gene silencing [73, 74]. Among others, EZH2 mediates transcriptional silencing of the tumor-suppressor gene E-cadherin [75]. This makes it a potential biomarker for disease progression.

## 7. Conclusion

With the recent used criteria for the prediction of low-risk PCa, still a substantial number of patients that need active treatment are missed. Hence, there is an urgent need for valid serum-,urine-, and tissue-based PCa biomarkers to distinguish insignificant PCa from those cancers that are inclined to progress to a more aggressive tumor and therefore, need active treatment. Albeit tissue markers seem promising in predicting disease progression, the detection of a robust marker in blood or urine will be the source of preference as it is the most ideal noninvasive approach.

Despite recent improvements in identification and the discovery of novel PCa biomarkers, it is still a long way to use these markers in a clinical setting. Furthermore, the use of a biomarker combination panel needs to be considered, to increase diagnostic accuracy and better manage low-risk PCa protocols. 
